# Supplementary material for: Identification of Molecular Markers Associated with Prostate Cancer Subtypes: An Integrative Bioinformatics Approach
Source: Biomolecules. 2024 Jan 10;14(1):87. doi: 10.3390/biom14010087 (PMC10813078; doi:10.3390/biom14010087)
Supplement: Supplementary file 1 [file biomolecules-14-00087-s001.zip › Supplementary file S1.pdf]

Supplementary Material

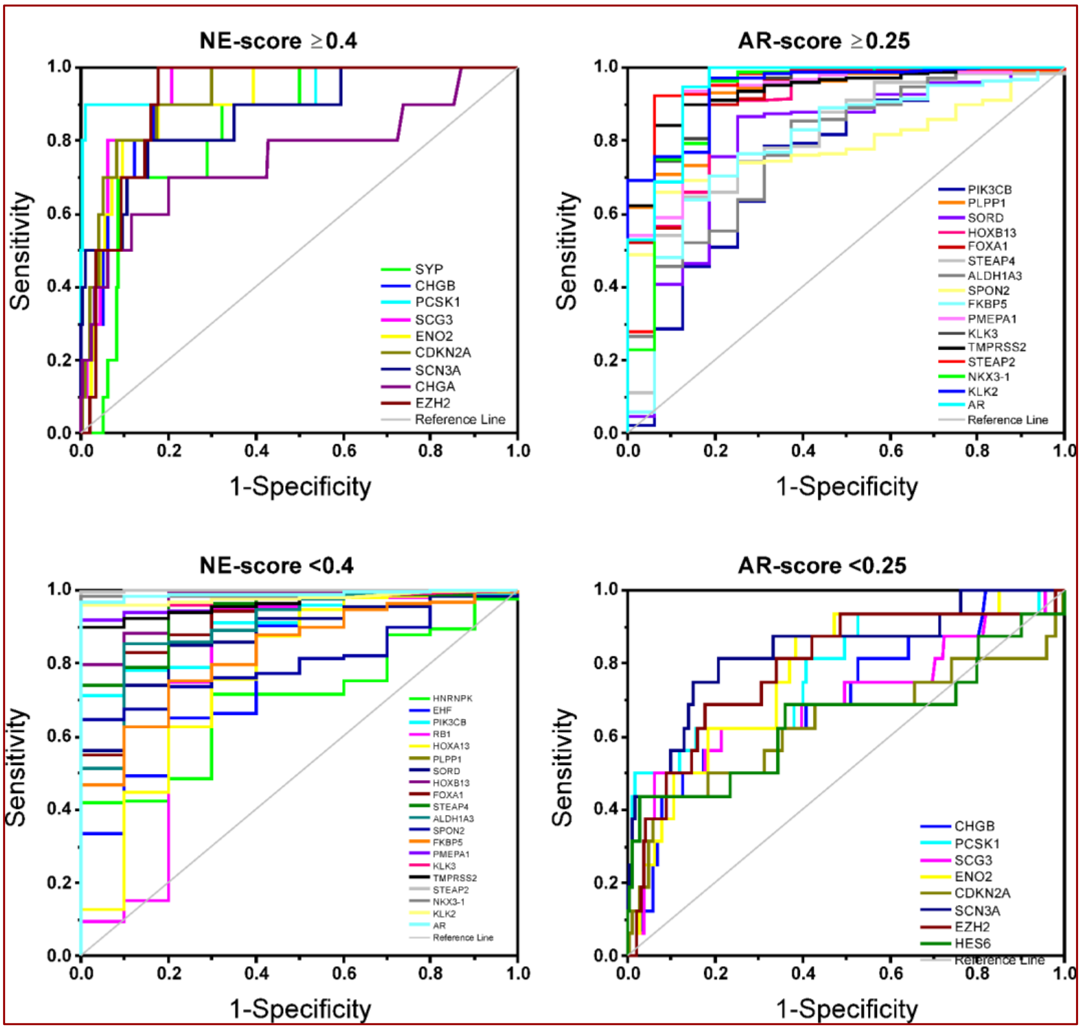

Figure S1. ROC curves performed to evaluate the diagnostic efficiency in discriminating between AR positive and NE positive CRPC samples by SU2C/PCF study (208 patients). Only significant AUC values >0.6 (Asymptotic Probability < 0.05) are reported.

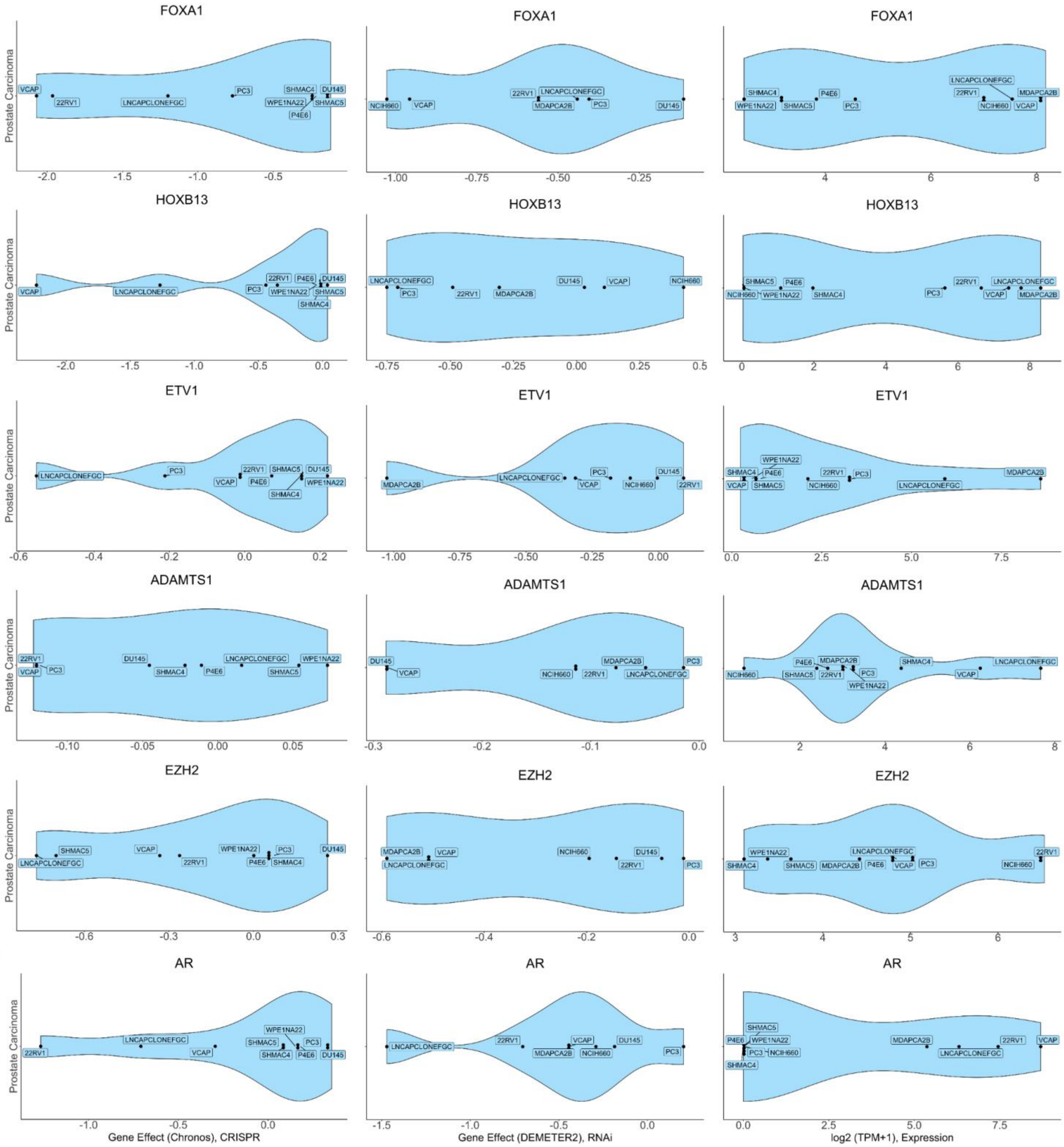

Figure S2. Violin plots showing the Gene effect scores from CRISPR (left panel) and RNAi (middle panel) experiments, and log2 (TPM+1) expression values (right panel) of FOXA1, HOXB13, ETV1, ADAMTS1, EZH2 and AR genes in androgen-sensitive PCa (LNCaP, MDAPC2A, MDAPC2A, P4E6) or CRPC (22Rv1, VCaP) cell lines, and androgen-insensitive PCa (SHMAC4, SHMAC5) or CRPC (PC3, DU145, NCIH660) cell line.

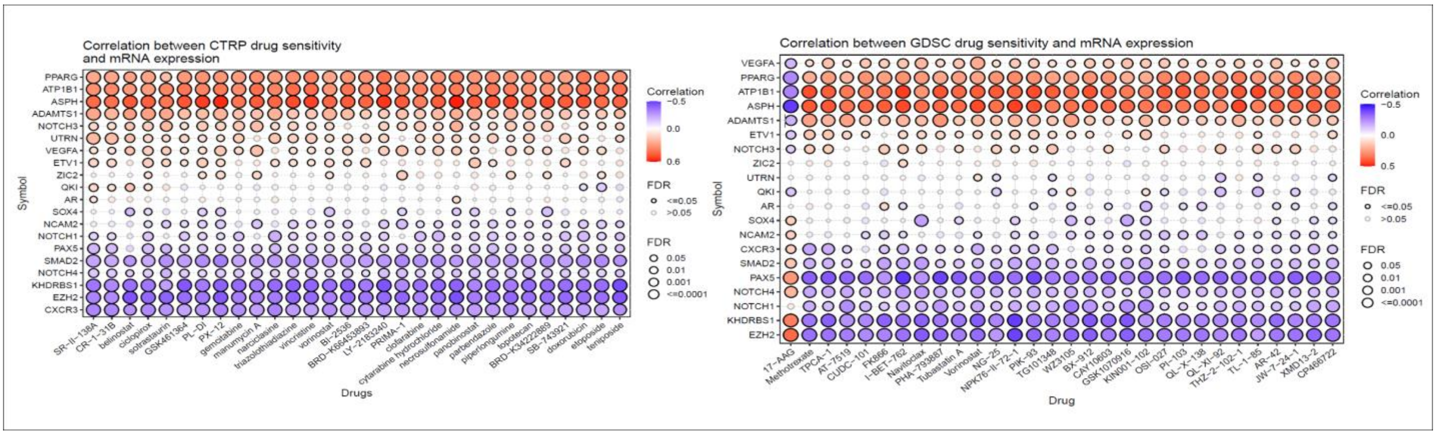

Figure S3. Correlation matrix between over-expressed PCa-gene involved in PCa progression to metastatic hormone sensitive (mPCa) or castration resistant (mCRPC) phenotypes and the sensitivity of CTRP (left) and GDSC (right) drugs in pan-cancer

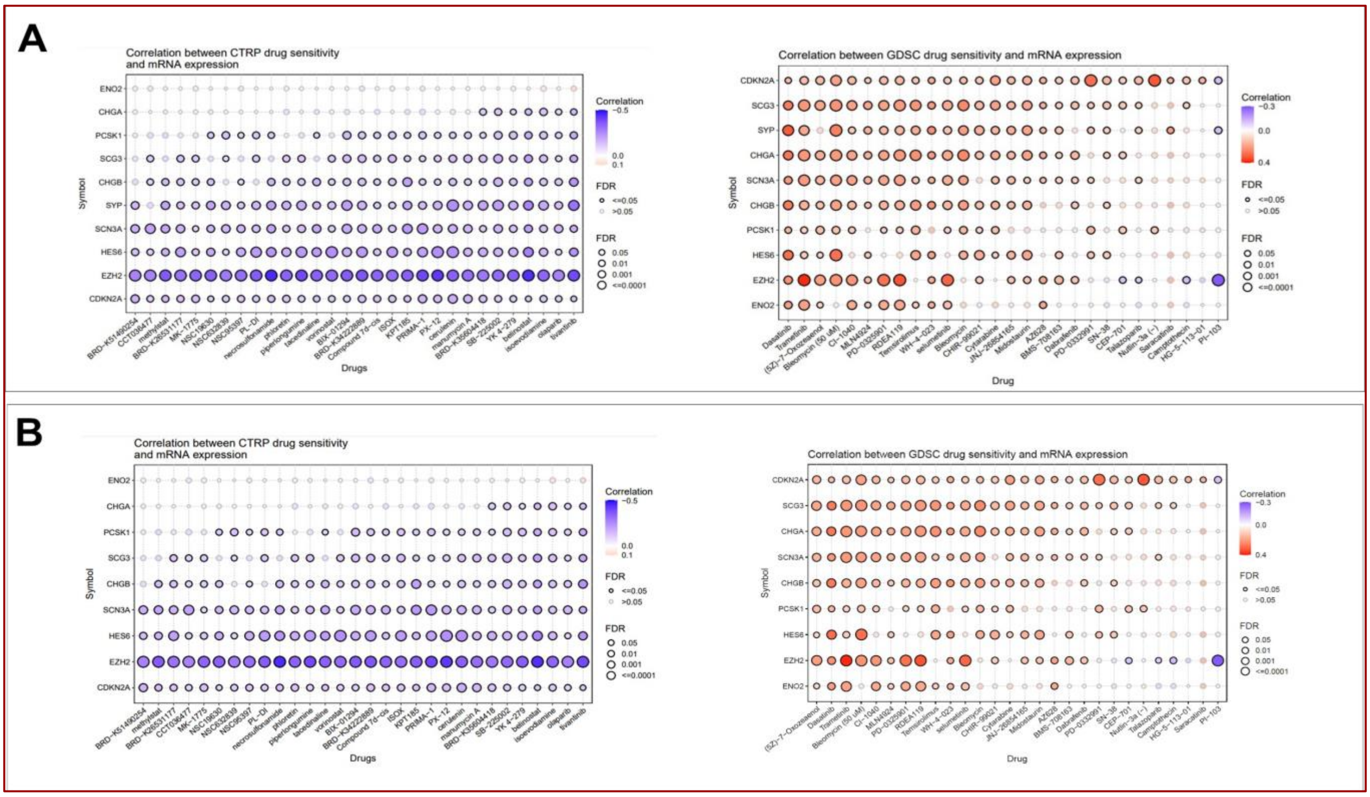

Figure S4. Correlation matrix between CRPC-gene set over-expressed in Adeno-CRPC (A) or in NE-CRPC (B) and the sensitivity of CTRP (left) and GDSC (right) drugs in pan-cancer.

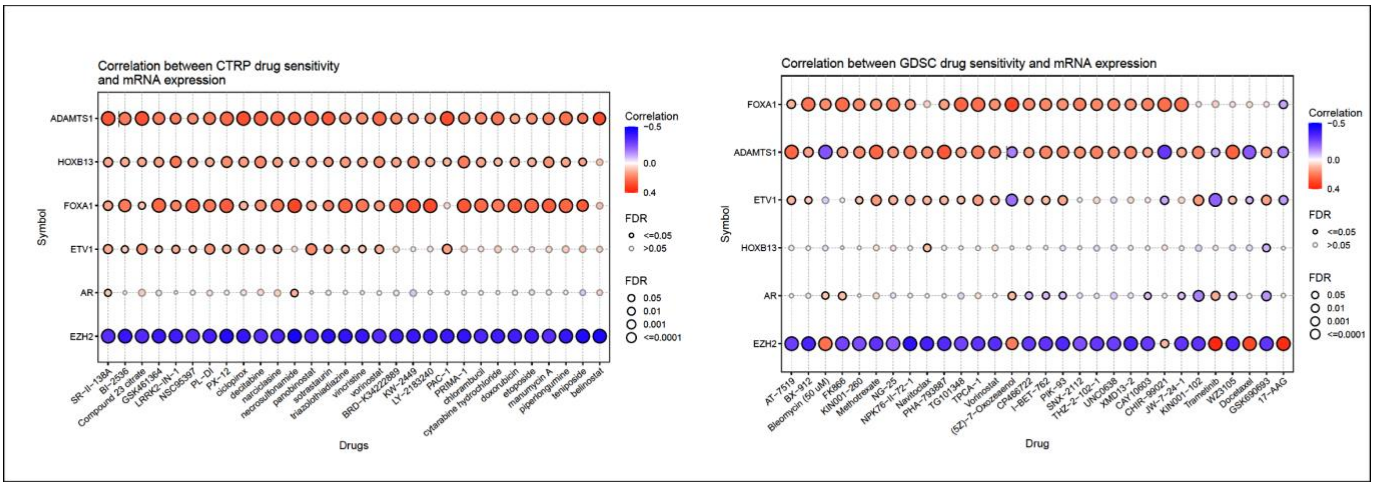

Figure S5. Correlation matrix between essential genes for the survival of PCa and CRPC cells and the sensitivity of CTRP (left) and GDSC (right) drugs in pan-cancer.

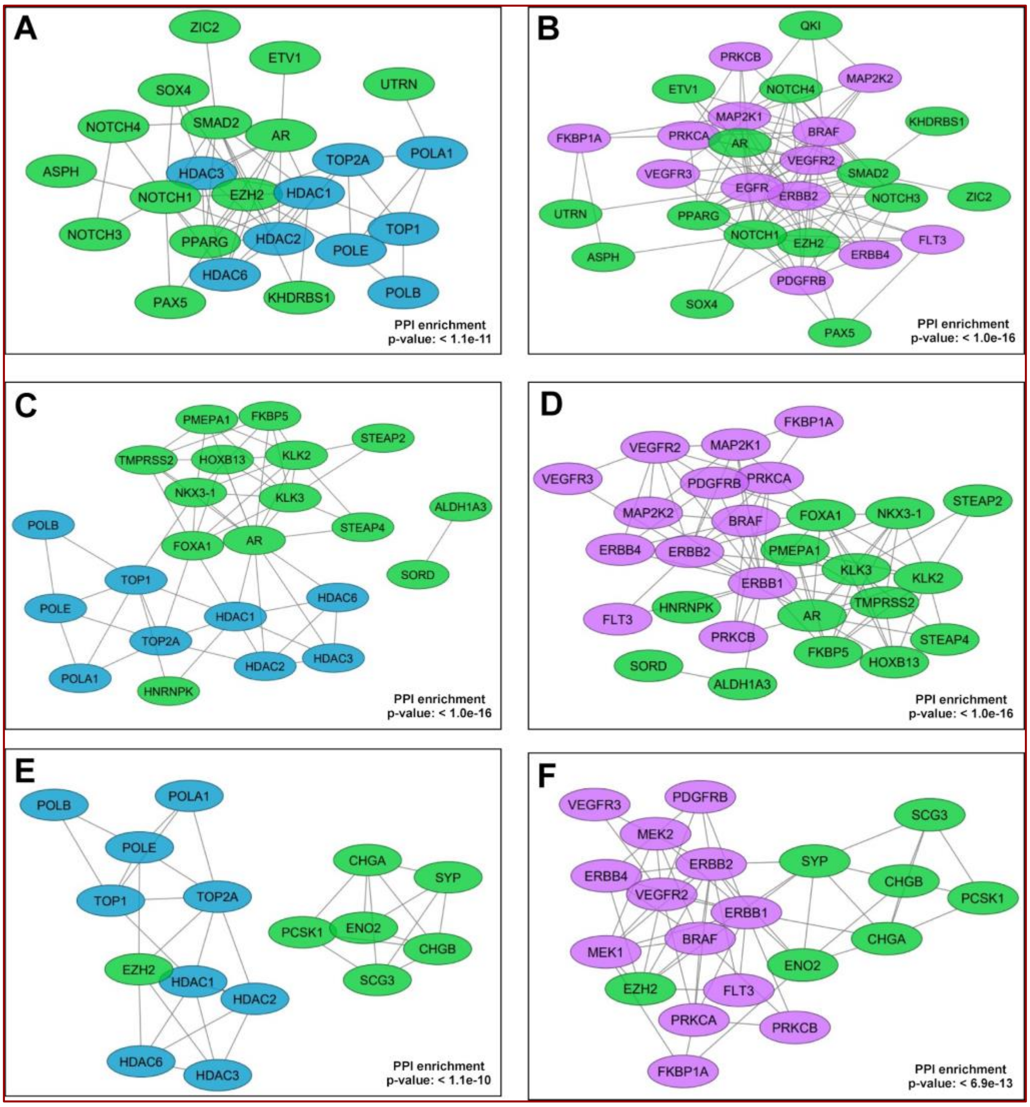

Figure S6. PPI networks showing the connections between the upregulated PCa (A, B), CRPC AR+ (C, D) and CRPC AR- (E, F) genes and the genes involved in the regulation of epigenetic transcription (A, C, E) or MAP kinase signal transduction (B, D, F) targeted by drugs reported in Table S11. The networks were obtained by using the STRING database and edited through the Cytoscape software (v3.9.9). The enrichment p-values are shown in each network box and indicate that these proteins have more interactions than expected and are, thus, biologically connected. Colour node: Green: PCa or CRPC gene set; Blue: epigenetic transcription regulation gene; Violet: MAP kinase signal transduction gene.

A

Up regulated in PCa gene set	Uniprot AC	Gene name
	Q9UHI8	ADAMTS1
	P10275	AR
	Q12797	ASPH
	P05026	ATP1B1
	P49682	CXCR3
	P50549	ETV1
	Q15910	EZH2
	Q07666	KHDRBS1
	O15394	NCAM2
	P46531	NOTCH1
	Q9UM47	NOTCH3
	Q99466	NOTCH4
	Q02548	PAX5
	P37231	PPARG
	Q96PU8	QKI
	Q15796	SMAD2
	Q06945	SOX4
	P46939	UTRN
	P15692	VEGFA
	O95409	ZIC2
	**O02705**	**HSP90AA1**

B

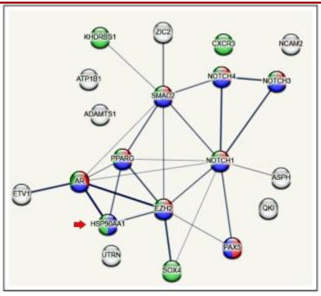

ENRICHMENT REACTOME PATHWAYS						
colour code	#term ID	term description	false discovery rate	observed gene count	background gene count	strength
RED	HSA-212436	Generic Transcription Pathway	0.0038	8	1215	0.81
BLUE	HSA-74160	Gene expression (Transcription)	0.0023	9	1476	0.78
GREEN	HSA-162582	Signal Transduction	0.0030	11	2540	0.63

C

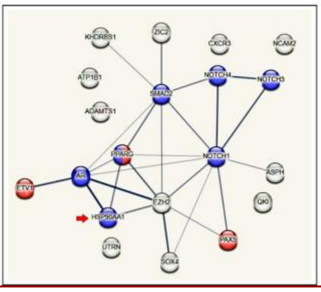

ENRICHMENT KEGG PATHWAYS						
colour code	#term ID	term description	false discovery rate	observed gene count	background gene count	strength
BLUE	hsa05200	Pathways in cancer	0.00017	7	515	1.13
RED	hsa05202	Transcriptional misregulation in cancer	0.0331	3	171	1.24

Figure S7. PPI networks showing the connections between the upregulated genes of the PCa gene set (A) and HSP90 (red arrow). The proteins involved in significantly enriched Reactome (B) or KEGG (C) pathways are highlighted. The networks and the enrichment results were obtained by using the STRING database.

Table S1. CRPC datasets used in this study

Ref.	# Genes	Gene list	CRPC phenotype	Dataset
[26]	8	AR, FOXA1, HOXB13, KLK3, NKX3-1, STEAP2, TMPRSS2, KLK2	AR driven,<br>non-AR driven	GSE101607
[29]	38	AR, FOXA1, AKT1, TP53, AMER1, APC, ATM, BRAF, BRCA1, BRCA2, CCND1, CDK4, CDKN1B, CDKN2A, CDKN2B, CHD1, CTNNB1, ERF, ETS1, FANCA, KRAS, MLH1,<br>MSH2, NCOR1, NCOR2, PIK3CA, PIK3CB, PIK3R1, PTEN, RAD51B, RAD51C, RAF1, RB1, RNF43, RSPO2, SPOP, ZBTB16, ZNRF3	metastatic	cBioPortal<br>Metastatic Prostate Adenocarcinoma (SU2C/PCF Dream Team, Cell 2015)
[27]	306	Supplementary table 5 from Harrison et al. paper: Meta genes with outlier expression in 60-80% (9-12) of 15 high-grade NEPC patients<br>178 Up-genes<br>128 Down-genes	neuroendocrine,<br>small cell	GSE104786
[28]	20	Up in AR+: KLK3, KLK2, TMPRSS2, FKBP5, NKX3-1, PPAP2A, PMEPA1, PART1, ALDH1A3, STEAP4<br>Up in NE+: SYP, CHGA, CHGB, ENO2, CHRNB2, SCG3, SCN3A, PCSK1, ELAVL4, NKX2-1	AR driven,<br>non-AR driven,<br>neuroendocrine	GSE77930

Table S2. List of the 32 proteins identified in previous papers by *in vitro* models.

Uniprot AC	Gene name	Protein name
O43707	ACTN4	Alpha-actinin-4
Q9UHI8	ADAMTS1	A disintegrin and metalloproteinase with thrombospondin motifs 1
P31749	AKT1	RAC-alpha serine/threonine-protein kinase
P51648	ALDH3A2	Aldehyde dehydrogenase family 3 member A2
P10275	AR	Androgen receptor
Q12797	ASPH	Aspartyl/asparaginyl beta-hydroxylase
P05026	ATP1B1	Sodium/potassium-transporting ATPase subunit beta-1
P27824	CANX	Calnexin
Q9NZC4	EHF	ETS homologous factor
P29320	EPHA3	Ephrin type-A receptor 3
P50549	ETV1	ETS translocation variant 1
P61978	HNRNPK	Heterogeneous nuclear ribonucleoprotein K
Q00839	HNRNPU	Heterogeneous nuclear ribonucleoprotein U
P31271	HOXA13	Homeobox protein Hox-A13
Q92826	HOXB13	Homeobox protein Hox-B13
P04792	HSPB1	Heat shock protein beta-1
P12268	IMPDH2	Inosine-5'-monophosphate dehydrogenase 2
Q07666	KHDRBS1	KH domain-containing, RNA-binding, signal transduction-associated protein 1
P07288	KLK3	Prostate-specific antigen
Q16539	MAPK14	Mitogen-activated protein kinase 14
P10242	MYB	Transcriptional activator Myb
O15394	NCAM2	Neural cell adhesion molecule 2
P09874	PARP1	Poly [ADP-ribose] polymerase 1
Q02548	PAX5	Paired box protein Pax-5
P30086	PEBP1	Phosphatidylethanolamine-binding protein 1
P37231	PPARG	Peroxisome proliferator-activated receptor gamma
Q96PU8	QKI	Protein quaking
Q00796	SORD	Sorbitol dehydrogenase
Q9BUD6	SPON2	Spondin-2
P46939	UTRN	Utrophin
P17861	XBP1	X-box-binding protein 1
O95409	ZIC2	Zinc finger protein ZIC 2

Table S3. Results obtained with Gene Set Enrichment Analysis (GSEA) using the 32 gene set as query genes. The Hallmark (H), chemical and genetic perturbations (CGP) and canonical pathways (CP) collections in MSigDB were evaluated.

# Genes in Gene Set (K)	Description	# Genes in Overlap (k)	k/K	p-value	FDR q-value	relevant gene in process or pathway regulation
128	Genes expressed in at least one prostate cancer cell line but not in normal prostate epithelial cells or stromal cells	7	0.0547	3.50E-12	1.20E-08	
101	Genes defining response to androgens.	4	0.0396	7.54E-07	3.77E-05	AR
53	Regulation of Androgen receptor activity	4	0.0755	5.53E-08	7.35E-05	AR
21	p38 signaling mediated by MAPKAP kinases	3	0.1429	4.04E-07	2.69E-04	MAPK14
200	Genes defining late response to estrogen.	4	0.02	1.14E-05	2.84E-04	
36	Adrenergic Pathway	3	0.0833	2.16E-06	7.79E-04	
37	p38 MAPK Pathway	3	0.0811	2.34E-06	7.79E-04	MAPK14
309	Genes down-regulated in LNCaP cells by overexpression of SOX4 [GeneID=6659] and up-regulated by its RNAi knockdown.	5	0.0162	2.31E-06	9.91E-04	SOX4
44	FOXA1 transcription factor network	3	0.0682	3.98E-06	1.06E-03	FOXA1
48	Notch-mediated HES/HEY network	3	0.0625	5.19E-06	1.15E-03	HES/HEY/NOTCH
169	Pubertal genes up-regulated by TGFB1 [GeneID=7040].	4	0.0237	5.86E-06	1.26E-03	TGFB1
1024	Genes down-regulated in PC3 cells (prostate cancer) after knockdown of EZH2 [GeneID=2146] by RNAi.	7	0.0068	5.53E-06	1.26E-03	EZH2
61	Coregulation of Androgen receptor activity	3	0.0492	1.07E-05	2.04E-03	AR
65	IL4-mediated signaling events	3	0.0462	1.30E-05	2.16E-03	IL4
76	VEGF signaling pathway	3	0.0395	2.08E-05	3.11E-03	VEGF
89	Prostate cancer	3	0.0337	3.34E-05	3.11E-03	
11	Amplification hot spot 29: colocolized fragile sites and cancer genes in the 7p22-p13 region.	2	0.1818	2.57E-05	3.33E-03	
86	Genes up-regulated in LNCaP cells (prostate cancer) in response to synthetic androgen R1881 [PubChem=13766].	3	0.0349	3.02E-05	3.45E-03	
200	Genes up-regulated through activation of mTORC1 complex.	3	0.015	3.67E-04	3.67E-03	MTOR
200	A subgroup of genes regulated by MYC - version 1 (v1).	3	0.015	3.67E-04	3.67E-03	MYC
975	Expressed genes associated with high-confidence PAX3-FOXO1 sites with enhancers in primary tumors and cell lines	6	0.0062	5.08E-05	4.84E-03	PAX3/FOXO1
328	Pathways in cancer	4	0.0122	7.82E-05	4.85E-03	
323	Genes from common genomic gains observed in a meta analyis of copy number alterations across a panel of different cancer cell lines and tumor	4	0.0124	7.37E-05	6.33E-03	
22	Apoptotic Signaling in Response to DNA Damage	2	0.0909	1.08E-04	8.85E-03	
24	Regulation of eIF4e and p70 S6 Kinase	2	0.0833	1.28E-04	8.85E-03	EIF4E/RPS6KB1
27	Transcription factor CREB and its extracellular signals	2	0.0741	1.63E-04	8.85E-03	CREB

Continued from Table S3						
# Genes in Gene Set (K)	Description	# Genes in Overlap (k)	k/K	p-value	FDR q-value	relevant gene in process or pathway regulation
84	Genes involved in Regulation of mRNA Stability by Proteins that Bind AU-rich Elements	3	0.0357	2.81E-05	9.48E-03	
466	Genes involved in Hemostasis	5	0.0107	1.69E-05	9.48E-03	
30	Retinoic acid receptors-mediated signaling	2	0.0667	2.02E-04	1.30E-02	
31	Nongenotropic Androgen signaling	2	0.0645	2.16E-04	1.30E-02	
31	CD40/CD40L signaling	2	0.0645	2.16E-04	1.30E-02	CD40/CD40L
40	p38 MAPK Signaling Pathway	2	0.05	3.60E-04	1.30E-02	MAPK14
432	Expressed genes associated with super-high-confidence PAX3-FOXO1 sites with highly-recurrent enhancers in primary tumors and cell lines	4	0.0093	2.25E-04	1.40E-02	PAX3/FOXO1
17	Genes involved in Destabilization of mRNA by KSRP	2	0.1176	6.35E-05	1.43E-02	KSRP
34	IL2 signaling events mediated by PI3K	2	0.0588	2.60E-04	1.44E-02	IL2
38	FAS (CD95) signaling pathway	2	0.0526	3.25E-04	1.66E-02	CD95
38	Signaling mediated by p38-alpha and p38-beta	2	0.0526	3.25E-04	1.66E-02	
102	Toll-like receptor signaling pathway	2	0.0196	6.49E-04	1.69E-02	TLR2
194	Cell cycle genes with peak expression in G1/S check point.	3	0.0155	3.36E-04	1.83E-02	
43	CXCR3-mediated signaling events	2	0.0465	4.17E-04	1.91E-02	CXCR3
45	Integrin-linked kinase signaling	2	0.0444	4.56E-04	2.02E-02	
47	IL6-mediated signaling events	2	0.0426	4.98E-04	2.13E-02	IL6
49	Genes involved in Nuclear Receptor transcription pathway	2	0.0408	5.41E-04	2.25E-02	
50	Angiopoietin receptor Tie2-mediated signaling	2	0.04	5.64E-04	2.27E-02	TEK
253	Genes down-regulated by MYC according to the MYC Target Gene Database.	3	0.0119	7.27E-04	2.68E-02	MYC
57	Thromboxane A2 receptor signaling	2	0.0351	7.32E-04	2.78E-02	TBXA2R
59	p53 pathway	2	0.0339	7.84E-04	2.89E-02	TP53
79	Direct targets of PRDM5	2	0.0253	2.62E-04	3.00E-02	PRDM5
63	Endothelins	2	0.0317	8.93E-04	3.08E-02	EDN1/EDN2/ EDN3
65	BCR signaling pathway	2	0.0308	9.51E-04	3.08E-02	BCR
65	Regulation of retinoblastoma protein	2	0.0308	9.51E-04	3.08E-02	RB1
65	Fas Signaling Pathway	2	0.0308	9.51E-04	3.08E-02	CD95
284	Genes involved in Metabolism of mRNA	3	0.0106	1.01E-03	3.21E-02	
69	Signaling events mediated by VEGFR1 and VEGFR2	2	0.029	1.07E-03	3.31E-02	VEGFR1/VEGFR2
144	Genes down-regulated in response to ultraviolet (UV) radiation.	2	0.0139	4.55E-03	3.79E-02	
267	MAPK signaling pathway	3	0.0112	8.49E-04	3.95E-02	
79	Fc epsilon RI signaling pathway	2	0.0253	1.40E-03	4.23E-02	FCER1G
82	Glucocorticoid receptor regulatory network	2	0.0244	1.51E-03	4.35E-02	NR3C1
82	Regulation of nuclear SMAD2/3 signaling	2	0.0244	1.51E-03	4.35E-02	SMAD2/SMAD3
84	C-MYB transcription factor network	2	0.0238	1.58E-03	4.37E-02	MYB
330	Genes involved in Metabolism of RNA	3	0.0091	1.56E-03	4.37E-02	

Table S4. GEPIA analysis results of relevant genes in process or pathway regulation reported in Supplementary Table 3 using the TCGA-Prostate Cancer dataset (PRAD).

		ANOVA analysis for tumors vs paired normal human prostate sample *		Disease free survival**	
**GENE NAME**	**PROTEIN NAME**	**Log2 FC**	**adjP**	**Logrank**	**HR (high)**
CD40	CD40 molecule	-1.6	7.05E-43		
CXCR3	C-X-C motif chemokine receptor 3			1.0E-02	1.8
EDN3	endothelin 3	-1.7	7.5E-38	5.4E-03	0.6
EZH2	enhancer of zeste 2 polycomb repressive complex 2 subunit			4.0E-04	2.2
FOXA1	forkhead box A1	1.4	3.7E-55		
HES6	hes family bHLH transcription factor 6			4.8E-02	1.5
IL4	interleukin 4	-1.2	1.2E-35		
MTOR	mechanistic target of rapamycin			2.7E-02	0.62
NOTCH1	notch 1	-1.4	1.51E-32		
NOTCH3	notch 3			4.9E-03	1.8
NOTCH4	notch 4	-1.8	5.1E-63	2.8E-02	1.6
PRDM5	PR/SET domain 5	-1.1	1.19E-37		
SMAD2	SMAD family member 2	-1.2	1.53E-32		
SOX4	SRY-box 4	1.3	1.6E-48		
TGFB1	transforming growth factor beta 1	-1.6	1.61E-32	1.2E-04	2.3
TLR2	toll like receptor 2	-1.1	5.63E-37		
TP53	tumor protein p53	3.7	0.0014	4.5E-02	0.65
VEGFA	vascular endothelial growth factor A	-1.4	1.54E-8		

* selection parameters: |log2fold-change| ≥1 and adjusted P value < 0.05

** selection parameters: Group cutoff, median and log rank < 0.05

Table S5. The 50-gene set list with correspondent protein name, association with androgen receptor retrieved from literature and disease term obtained using Gene Set to Diseases web platform (http://cbdm.uni-mainz.de/geneset2diseases).

Uniprot	GENE NAME	PROTEIN NAME	Association with Androgen Receptor [34,38-45]	Prostatic Neoplasms	Prostatic Neoplasms, Castration-Resistant	Neoplasm Invasiveness	Neoplasm Metastasis	Carcinogenesis
O43707	ACTN4	Alpha-actinin-4	AR coactivator/ corepressor			yes	yes	
Q9UHI8	ADAMTS1	A disintegrin and metalloproteinase with thrombospondin motifs 1						
P31749	AKT1	RAC-alpha serine/threonine-protein kinase	AR corepressor, direct interaction	yes	yes	yes	yes	yes
P51648	ALDH3A2	Fatty aldehyde dehydrogenase						
P10275	AR	Androgen receptor	AR controlled, direct interaction	yes	yes	yes	yes	
Q12797	ASPH	Aspartyl/asparaginyl beta-hydroxylase						
P05026	ATP1B1	Sodium/potassium-transporting ATPase subunit beta-1	AR controlled					
P27824	CANX	Calnexin						
P25942	CD40	CD40 molecule						
P49682	CXCR3	C-X-C motif chemokine receptor 3				yes		
P14138	EDN3	endothelin 3						
Q9NZC4	EHF	ETS homologous factor		yes		yes		
P29320	EPHA3	Ephrin type-A receptor 3	AR controlled					
P50549	ETV1	ETS translocation variant 1	AR coregulator, direct interaction, AR controlled	yes		yes		
Q15910	EZH2	enhancer of zeste 2 polycomb repressive complex 2 subunit	AR controlled	yes	yes	yes	yes	yes
P55317	FOXA1	forkhead box A1	AR coactivator/ corepressor; direct interactor	yes		yes	yes	

Continued from Table S5								
**Uniprot**	**GENE NAME**	**PROTEIN NAME**	**Association with Androgen Receptor [34,38-45]**	**Prostatic Neoplasms**	**Prostatic Neoplasms, Castration-Resistant**	**Neoplasm Invasiveness**	**Neoplasm Metastasis**	**Carcinogenesis**
Q96HZ4	HES6	hes family bHLH transcription factor 6	AR controlled					
P61978	HNRNPK	Heterogeneous nuclear ribonucleoprotein K	AR coregulator			yes	yes	
Q00839	HNRNPU	Heterogeneous nuclear ribonucleoprotein U						
P31271	HOXA13	Homeobox protein Hox-A13						
Q92826	HOXB13	Homeobox protein Hox-B13	AR coactivator, direct interaction , AR controlled	yes		yes	yes	
P04792	HSPB1	Heat shock protein beta-1; HSP27	AR coactivator, direct interaction , AR controlled			yes		
P05112	IL4	interleukin 4						
P12268	IMPDH2	Inosine-5-monophosphate dehydrogenase 2						
Q07666	KHDRBS1	KH domain-containing, RNA-binding, signal transduction-associated protein 1; Sam68	AR coactivator, direct interaction					
P07288	KLK3	Prostate-specific antigen	AR controlled	yes	yes		yes	
Q16539	MAPK14	p38MAPK				yes	yes	yes
P42345	MTOR	mechanistic target of rapamycin		yes		yes	yes	yes
P10242	MYB	V-myb myeloblastosis viral oncogene homologue	AR controlled			yes		
O15394	NCAM2	Neural cell adhesion molecule 2						
P46531	NOTCH1	notch 1				yes	yes	yes
Q9UM47	NOTCH3	notch 3						yes
Q99466	NOTCH4	notch 4				yes		

Continued from Table S5								
**Uniprot**	**GENE NAME**	**PROTEIN NAME**	**Association with Androgen Receptor [34,38-45]**	**Prostatic Neoplasms**	**Prostatic Neoplasms, Castration-Resistant**	**Neoplasm Invasiveness**	**Neoplasm Metastasis**	**Carcinogenesis**
P09874	PARP1	Poly [ADP-ribose] polymerase 1	AR controlled	yes				
Q02548	PAX5	Paired box protein Pax-5						
P30086	PEBP1	Raf kinase inhibitor protein (RKIP)		yes		yes	yes	
P37231	PPARG	Peroxisome proliferator-activated receptor gamma	AR coregulator, AR controlled					
Q9NQX1	PRDM5	PR/SET domain 5						
Q96PU8	QKI	Quaking						
Q15796	SMAD2	SMAD family member 2				yes	yes	
Q00796	SORD	Sorbitol dehydrogenase	AR controlled					
Q06945	SOX4	SRY-box 4	AR controlled	yes		yes	yes	
Q9BUD6	SPON2	Spondin-2	AR controlled					
P01137	TGFB1	transforming growth factor beta 1				yes	yes	yes
O60603	TLR2	toll like receptor 2						
P04637	TP53	tumor protein p53	AR corepressor; indirect interactor			yes	yes	yes
P46939	UTRN	Utrophin						
P15692	VEGFA	vascular endothelial growth factor A				yes	yes	yes
P17861	XBP1	X-box binding protein 1						
O95409	ZIC2	Zic family member 2	AR controlled					

Table S6. Log2fold-change values of 50-gene set obtained by GEO2R analysis. The eight GEO datasets reporting mRNA data were analyzed using the expression levels thresholds |log2fold-change| ≥1 and adjusted P<0.05

	**mPCa vs PCa**			**mCRPC vs PCa**				
GENE NAME	GSE3325	GSE3933	GSE68882	GSE32269	GSE6811	GSE70770	GSE6752	GSE35988
ACTN4								
ADAMTS1	-3.2						1.5	
AKT1								
ALDH3A2				-1.4	-1.4		-2.5	
AR		1.5		2.4			2.0	1.5
ASPH	3.6			1.1				
ATP1B1	1.4							
CANX								
CD40	-1.6						-2.0	
CXCR3							1.1	
EDN3	-1.9	-1.1					-1.8	
EHF	-1.8						-2.2	
EPHA3	-3.7						-2.4	
ETV1	2						-1.1	
EZH2	2.5		1.6	1.8				
FOXA1	-1.0					-1.6	-1.2	
HES6								
HNRNPK								
HNRNPU								
HOXA13	-2.5							
HOXB13	-2.5			-1.4	-1.7			
HSPB1								
IL4					-1.4		1.1	
IMPDH2								
KHDRBS1	1.4							
KLK3	3.9		-2.1	-1.6		-2.6	-3.8	
MAPK14								
MTOR								
MYB								
NCAM2	1.6						1.2	
NOTCH1						1.0		
NOTCH3			1.1	1.2			1.0	
NOTCH4							1.0	
PARP1								
PAX5	1.5							
PEBP1								
PPARG	2.6		1.2					
PRDM5	-2.6							
QKI	-1.7	1.3		1.2				

Continued from Table S6								
	**mPCa vs PCa**			**mCRPC vs PCa**				
GENE NAME	GSE3325	GSE3933	GSE68882	GSE32269	GSE6811	GSE70770	GSE6752	GSE35988
SMAD2	2.5						-1.5	
SORD	-1.9		-2.3	-2.5		-2.4	-2.2	
SOX4	1.6							
SPON2						-1.7	-1.4	
TGFB1								
TLR2								
TP53	-2.4							
UTRN	1							
VEGFA	3.7						1.3	
XBP1		-1.2	-1.2	-1.4	-1.8	-1.5	-1.4	
ZIC2	6.0						4.3	

Table S7. The PCa-gene set characteristics

Gene name	Uniprot AC	Protein name	Association with Androgen Receptor activity [34,38-45]
ADAMTS1	Q9UHI8	A disintegrin and metalloproteinase with thrombospondin motifs 1	
ALDH3A2	P51648	Aldehyde dehydrogenase family 3 member A2	
AR	P10275	Androgen receptor	AR controlled, direct interactor
ASPH	Q12797	Aspartyl/asparaginyl beta-hydroxylase	
ATP1B1	P05026	Sodium/potassium-transporting ATPase subunit beta-1	AR controlled
CD40	P25942	CD40 molecule	
CXCR3	P49682	C-X-C motif chemokine receptor 3	
EDN3	P14138	endothelin 3	
EHF	Q9NZC4	ETS homologous factor	
EPHA3	P29320	Ephrin type-A receptor 3	AR controlled
ETV1	P50549	ETS translocation variant 1	AR coregulator, direct interactor, AR controlled
EZH2	Q15910	enhancer of zeste 2 polycomb repressive complex 2 subunit	AR controlled
FOXA1	P55317	forkhead box A1	AR coactivator/ corepressor; direct interactor
HOXA13	P31271	Homeobox protein Hox-A13	
HOXB13	Q92826	Homeobox protein Hox-B13	AR coactivator, direct interactor, AR controlled
KHDRBS1	Q07666	KH domain-containing, RNA-binding, signal transduction-associated protein 1; Sam68	AR coactivator, direct interactor
KLK3	P07288	Prostate-specific antigen	AR controlled
NCAM2	O15394	Neural cell adhesion molecule 2	
NOTCH1	P46531	notch 1	
NOTCH3	Q9UM47	notch 3	
NOTCH4	Q99466	notch 4	
PAX5	Q02548	Paired box protein Pax-5	
PPARG	P37231	Peroxisome proliferator-activated receptor gamma	AR coregulator, AR controlled
PRDM5	Q9NQX1	PR/SET domain 5	
QKI	Q96PU8	Quaking	
SMAD2	Q15796	SMAD family member 2	
SORD	Q00796	Sorbitol dehydrogenase	AR controlled
SOX4	Q06945	SRY-box 4	AR controlled
SPON2	Q9BUD6	Spondin-2	AR controlled
TP53	P04637	tumor protein p53	AR corepressor; indirect interactor
UTRN	P46939	Utrophin	
VEGFA	P15692	vascular endothelial growth factor A	
XBP1	P17861	X-box binding protein 1	
ZIC2	O95409	Zic family member 2	AR controlled

Table S8. The 34 PCa-gene set differential expression in metastatic versus primary PCa (MP1) and in mCRPC versus PCa (MP2) comparison, and their prognostic efficiency evaluated by ROC curve analysis.

	**Deregulated expression**		**Prognostic efficiency (ROC analysis)** [£]					
**Gene name**[#]	**mPCa vs PCa (MP1)**	**mCRPC vs PCa (MP2)**	**Disease-specific Survival status (dead with tumor)**[§]	**Disease free status (recurred/ progressed)** [§]	**Progression free status (recurred)** [§]	**New Neoplasm Event Post Initial Therapy (yes)** [§]	**Subgroup 1 (high recurrence risk)** [§]	**Metastasis presence**[$]
ADAMTS1	down		0.80943				0.65336	0.79028
ALDH3A2		down		0.67131			0.80643	0.89916
**AR** *	up	up						0.87746
ASPH	up	up						
ATP1B1*	up							
CD40	down	down					0.78931	
CXCR3		up						
EDN3	down	down	0.8377	0.61001			0.8823	
**EHF**	down	down						0.66171
EPHA3*	down	down		0.60871			0.7248	0.94134
ETV1*		down					0.6088	
**EZH2***	up	up			0.65711	0.64478	0.75452	0.86822
**FOXA1***	down	down						
**HOXA13**	down						0.60046	
**HOXB13***	down	down						
KHDRBS1*	up							
**KLK3***		down						0.68903
NCAM2	up	up						

Continued from Table S8								
	**Deregulated expression**		**Prognostic efficiency (ROC analysis)** [£]					
**Gene name**[#]	**mPCa vs PCa (MP1)**	**mCRPC vs PCa (MP2)**	**Disease-specific Survival status (dead with tumor)**[§]	**Disease free status (recurred/ progressed)** [§]	**Progression free status (recurred)** [§]	**New Neoplasm Event Post Initial Therapy (yes)** [§]	**Subgroup 1 (high recurrence risk)** [§]	**Metastasis presence**[$]
NOTCH1		up				0.63983		
NOTCH3	up	up			0.62246	0.6302		0.7947
NOTCH4		up						
PAX5	up							0.64002
PPARG*	up							
PRDM5	down						0.69758	0.89634
QKI		up						
SMAD2	up	down						
**SORD**	down	down					0.619	0.79952
SOX4*	up						0.68257	0.7047
**SPON2***		down	0.78852					
TP53*	down							
UTRN	up							
VEGFA	up	up						
XBP1	down	down					0.71105	0.85818
ZIC2*	up	up						0.71073

[#] In bold genes shared by Table S8
* AR-associated gene from Table S5
[£] Only significant AUC value >0.6 (Asymptotic Probability < 0.05) are reported
[§] From the Prostate Adenocarcinoma (PRAD-TCGA, PanCancer Atlas, 493 patient) study
[$] From the Prostate Adenocarcinoma (MSK, Cancer Cell 2010, 150 patients) study

Table S9. The CRPC-gene set characteristics

Gene name	Uniprot AC	Protein name	Association with Androgen Receptor Activity [34,38-45]
ALDH1A3	P47895	Aldehyde dehydrogenase family 1 member A3	AR controlled
AR	P10275	Androgen receptor	AR controlled, direct interactor
CDKN2A	Q9NXV6	CDKN2A-interacting protein	
CHGA	P10645	Chromogranin-A	
CHGB	P05060	Secretogranin-1	
EHF	Q9NZC4	ETS homologous factor	
ENO2	P09104	Gamma-enolase	
EZH2	Q15910	enhancer of zeste 2 polycomb repressive complex 2 subunit	AR controlled
FKBP5	Q13451	Peptidyl-prolyl cis-trans isomerase FKBP5	AR controlled;AR coactivator; indirect interactor
FOXA1	P55317	forkhead box A1	AR coactivator/ corepressor; direct interactor
HES6	Q96HZ4	hes family bHLH transcription factor 6	AR controlled
HNRNPK	P61978	Heterogeneous nuclear ribonucleoprotein K	AR coregulator
HOXA13	P31271	Homeobox protein Hox-A13	
HOXB13	Q92826	Homeobox protein Hox-B13	AR coactivator, direct interactor , AR controlled
KLK2	P20151	Kallikrein-2	AR controlled
KLK3	P07288	Prostate-specific antigen	AR controlled
NKX3-1	Q99801	Homeobox protein Nkx-3.1	AR controlled
PCSK1	P29120	Neuroendocrine convertase 1	
PIK3CB	P42338	Phosphatidylinositol 4,5-bisphosphate 3-kinase catalytic subunit beta isoform	AR coactivator
PLPP1	O14494	Phospholipid phosphatase 1	
PMEPA1	Q969W9	Protein TMEPAI	direct interactor
RB1	P06400	Retinoblastoma-associated protein	AR coactivator, direct interactor
SCG3	Q8WXD2	Secretogranin-3	
SCN3A	Q9NY46	Sodium channel protein type 3 subunit alpha	
SORD	Q00796	Sorbitol dehydrogenase	AR controlled
SPON2	Q9BUD6	Spondin-2	AR controlled
STEAP2	Q8NFT2	Metalloreductase STEAP2	
STEAP4	Q687X5	Metalloreductase STEAP4	
SYP	P08247	Synaptophysin	
TMPRSS2	O15393	Transmembrane protease serine 2	AR controlled

Table S10. The CRPC-gene list reporting differential expression evaluated in CRPC AR- vs CRPC AR+ and diagnostic efficiency (AUC) evaluated by ROC curve analysis

	Deregulated expression	Diagnostic efficiency (AUC[$]) from SU2C/PCF study (208 patients)						Diagnostic efficiency (AUC*) from neuroendocrine-PC study (49 patients)	
Gene name[#]	CRPC AR- vs CRPC AR+ (mean LogFC§)	AR score > 0.25 (AR+)	AR score < 0.25 (AR-)	NE score >0.4	NE score <0.4	CRPC NE (high expression level)	CRPC ADENO (high expression level)	CRPC NE (high expression level)	CRPC ADENO (high expression level)
CDKN2A	1.27		0.63216	0.92626		0.96355		NA	
CHGA	1.09			0.75429		0.78079		0.86765	
CHGB	3.89		0.71208	0.92273		0.8867		0.9549	
ENO2	1.93		0.77604	0.90505		0.92611		0.80686	
**EZH2***	0.70		0.78874	0.91717		0.90148		0.7951	
HES6	0.69		0.64941	0.93889		0.98818		0.91078	
PCSK1	2.86		0.78255	0.94394		0.99113		0.88333	
SCG3	2.81		0.69743	0.9298		0.933		0.85882	
SCN3A	1.20		0.83333	0.86869		0.97635		0.84706	
SYP	0.43			0.8358		0.867		0.9274	
ALDH1A3	-2.36	0.78743					0.61107		0.90098
**AR***	-3.76	0.94792			0.9904		0.65376		0.87647
**EHF**	-0.62	0.71549					0.67371		
FKBP5	-2.41	0.79329					0.67517		0.7098
**FOXA1***	-1.73	0.91178					0.70992		
HNRNPK	-0.39	0.69798					0.68171		
**HOXA13**	-1.00	0.68262							
**HOXB13***	-1.67	0.90511			0.96566		0.6385		0.80588
KLK2	-3.41	0.94336			0.97879				0.85294

Continued from Table S10									
	Deregulated expression	Diagnostic efficiency (AUC[$]) from SU2C/PCF study						Diagnostic efficiency (AUC[*]) from neuroendocrine-PC study	
Gene name[#]	CRPC AR- vs CRPC AR+ (mean LogFC§)	AR score > 0.25 (AR+)	AR score < 0.25 (AR-)	NE score >0.4	NE score <0.4	CRPC NE (high expression level)	CRPC ADENO (high expression level)	CRPC NE (high expression level)	CRPC ADENO (high expression level)
**KLK3**[*]	-2.76	0.94499			0.97879		0.69697		0.91471
NKX3-1[*]	-3.27	0.91764			0.9904				0.93333
PIK3CB	-0.83	0.7474							
PLPP1	-1.63	0.9209			0.98434		0.61795		0.82157
PMEPA1	-2.46	0.92611			0.97071		0.62971		0.70392
RB1	-0.94							NA	
**SORD**[*]	-1.64	0.7972					0.64398	NA	
**SPON2**	-2.40	0.78841							0.68824
STEAP2	-3.13	0.93717			0.99949				0.75882
STEAP4	-2.15	0.80534			0.93939				0.78627
TMPRSS2	-3.00	0.93848			0.96566		0.61266		0.71176

[#] In bold genes shared by Table S7
* AR-associated gene from Table S5
[$] Only significant AUC values >0.6 (Asymptotic Probability < 0.05) are reported
§ Average values across the GSE101607 and GSE77930 datasets

Table S11. FDA-approved drugs for neoplasms treatment.

Drug	CHEMBL ID	Target	Ref.*	Inhibited Process	Potential clinical application		
Belinostat^	CHEMBL408513	HDAC1	49	Epigenetic transcription regulation	PCa	Adeno-CRPC	
Panobinostat^	CHEMBL483254	HDAC1	50,51,52		PCa	Adeno-CRPC	
Vorinostat^	CHEMBL98	HDAC1, HDAC2, HDAC3, HDAC6	52,53		PCa	Adeno-CRPC	
Topotecan	CHEMBL2362989	TOP1	54			Adeno-CRPC	
Etoposide^	CHEMBL44657	TOP2A	55,56		PCa	Adeno-CRPC	
Teniposide^	CHEMBL452231	TOP2A				Adeno-CRPC	
Doxorubicin^	CHEMBL53463	TOP2A, DNA	57,58		PCa	Adeno-CRPC	
Cytarabine	CHEMBL803	DNA, RNA, DNA polymerase	59			Adeno-CRPC	NE- CRPC
Bleomycin^	CHEMBL403664	DNA	60				NE- CRPC
Midostaurin	CHEMBL608533	VEGFR2, SCFR, PKC, FLT3		MAP kinase signal transduction regulation			NE- CRPC
Axitinib	CHEMBL1289926	VEGFR3				Adeno-CRPC	
Dabrafenib	CHEMBL2028663	BRAF	61				NE- CRPC
Afatinib	CHEMBL1173655	ERBB1, ERBB2, ERBB4	62,63			Adeno-CRPC	
Dasatinib	CHEMBL1421	MEK1, MEK2	8,64,65, 66,52				NE- CRPC
Selumetinib	CHEMBL1614701	MEK1, MEK2					NE- CRPC
Trametinib^	CHEMBL2103875	MEK1, MEK2	67				NE- CRPC
Temsirolimus	CHEMBL1201182	FKBP1A	68,52				NE- CRPC
Methotrexate^	CHEMBL34259	DHFR	69	Folate metabolism	PCa	Adeno-CRPC	
Vincristine^	CHEMBL90555	TUBB1	70	Mitosis	PCa	Adeno-CRPC	
Ciclopirox^	CHEMBL1413	Iron chelator	71	Multiple pathways	PCa	Adeno-CRPC	

*References reporting preclinical and clinical studies for PCa and CRPC treatment

^ Potentially active on essential genes FOXA1, HOXB13, ETV1, ADMATS1, EZH2 and AR
